# High‐Precision 3D Doping of Fused Silica Glass Derived from Nanocomposites

**DOI:** 10.1002/adma.202511245

**Published:** 2025-08-03

**Authors:** Richard Prediger, Sebastian Kluck, Leonhard Hambitzer, Bastian E. Rapp, Silvio Tisato, Josephine N. Häberlein, Dorothea Helmer, Frederik Kotz‐Helmer

**Affiliations:** ^1^ Laboratory of Process Engineering, NeptunLab, Department of Microsystems Engineering (IMTEK) University of Freiburg 79110 Freiburg Germany; ^2^ Freiburg Materials Research Center (FMF) University of Freiburg 79104 Freiburg Germany; ^3^ Electrochemical Energy Systems, IMTEK, Department of Microsystems Engineering (IMTEK) University of Freiburg 79110 Freiburg Germany; ^4^ Freiburg Center for Interactive Materials and Bioinspired Technologies University of Freiburg 79110 Freiburg Germany; ^5^ Glassomer GmbH In den Kirchenmatten 54 79110 Freiburg Germany

**Keywords:** glass doping, multicomponent glasses, sintering, two‐photon polymerization

## Abstract

Glasses are utilized for their outstanding optical, mechanical, and thermal properties. However, conventional production methods mostly yield in glasses with uniform compositions and material properties. Here a novel lithographic approach is presented for high‐resolution 3D dopant integration at defined positions, which enables property modifications in specific regions. For this, a porous glass matrix derived from nanocomposites is shaped using 3D printing or injection molding. Using volumetric 3D printing like computed axial or two‐photon lithography, doping is performed within the porous glass using photocurable metal oxide precursors. The dopant is then permanently integrated within the glass during a final sintering step. The local integration of dopants like Ti^4+^, Co^2+^, Eu^3+^ or Tb^3+^ allow to selectively change the color, luminescence or refractive index within a 3D‐shaped glass with micron resolution. The process enables a wide range of novel applications from integrated optics and photonics to mass customization, anti‐counterfeiting, and information storage.

## Graphical abstract

The local integration of dopants enables the selective modification of color, luminescence or refractive index within a three‐dimensionally shaped glass with micron resolution. Following the fabrication of porous glass preforms and infusion with a dopant mixture, the dopant is immobilized by lithographic techniques and subsequently debinded and sintered. This enables to integrate functions in complex‐shaped glass components.

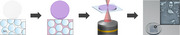

## 1 Introduction

Due to their high transparency, chemical and thermal resistance and durability, silicate‐based glasses have been widely used by mankind for thousands of years and remain among the most important materials for modern technologies.^[^ ^1^, ^2^ ^]^ In the early days, glasses were primarily used for simple containers or windows, however, their modern applications extend to functional roles in optics, photonics, sensors and information storage.^[^ ^2^, ^3^, ^4^, ^5^ ^]^ In these advanced applications, the function of a glass is defined by its shape as well as its internal material properties. In optical and photonic applications, for example, light guidance is determined by the shape of, e.g., a lens curvature but also influenced by the material properties like the refractive index or the dispersion.^[^ ^6^, ^7^ ^]^ Traditionally, on the macroscopic scale, glasses were manufactured from melts. While the shape can be subsequently modified using for example grinding or polishing, the internal material properties remain constant.^[^ ^8^ ^]^ On the microscale glasses, are mainly shaped using etching processes and local material property modifications are mostly limited to laser‐based approaches for changing the refractive index within the glass bulk.^[^ ^9^, ^10^, ^11^, ^12^, ^13^, ^14^ ^]^ These modifications are however slow and limited to only slight changes in the refractive index in the range of 10^−4^ to 10^−3^.^[^ ^12^, ^13^ ^]^

Recently a variety of indirect glass shaping methods have been introduced, which revolutionized the way glasses can be shaped. Fused silica glasses can be produced by shaping precursor materials via polymer processing methods, such as 3D printing or injection molding, followed by a heat treatment. The main indirect methods are based on particle‐based nanocomposites and sol‐gel based materials. Using particle based nanocomposites it was shown that fused silica glasses can be shaped using UV‐casting,^[^ ^15^, ^16^ ^]^ vat photopolymerization,^[^ ^17^, ^18^ ^]^ two‐photon polymerization (TPL),^[^ ^19^, ^20^ ^]^ fused deposition modeling,^[^ ^21^ ^]^ volumetric printing methods like computed axial lithography (CAL)^[^ ^22^ ^]^ or injection molding (IM).^[^ ^23^, ^24^ ^]^ Following shaping, these nanocomposites are debinded and sintered to full density fused silica glass. Furthermore, fused silica glass printing techniques using sol‐gel alkoxide precursors or hydrogen silsesquioxanes have been reported. These methods include vat photopolymerization,^[^ ^25^ ^]^ direct ink writing (DIW)^[^ ^26^, ^27^ ^]^ and TPL.^[^ ^20^, ^28^, ^29^, ^30^, ^31^ ^]^ Besides pure fused silica glasses, novel methods for the production of multicomponent glasses were demonstrated, which allow the material properties to be tailored to specific applications. In our previous work, we have already shown that printed or injection‐molded colored or fluorescent glasses can be produced by doping with different metal salts.^[^ ^17^, ^23^ ^]^ Therefore, the shaped nanocomposite were thermally debinded, yielding porous so‐called brown parts composed of silica nanoparticles. These were then soaked in the metal salt solutions and subsequently sintered. Furthermore, we have presented nanocomposites containing photocurable metal oxide precursors that enable UV‐casting as well as 3D printing using vat photopolymerization and high‐resolution TPL of multicomponent glasses.^[^ ^32^ ^]^ Using this approach, we demonstrated the fabrication of binary and ternary glasses containing TiO_2_, GeO_2_ or ZrO_2_. By adding these metal oxides, the refractive index and the coefficient of thermal expansion were significantly modified. Sol‐gel‐based methods for 3D printing of multicomponent glasses have also been demonstrated. Binary and ternary glasses were produced using vat photopolymerization,^[^ ^33^ ^]^ digital light processing^[^ ^34^ ^]^ and DIW.^[^ ^35^, ^36^ ^]^

However, these novel shaping methods only allow shaping of a glass with a homogeneous composition. First attempts to locally change the material properties were conducted by partially soaking a porous brown part with a salt solution, which resulted in luminescence after sintering.^[^ ^37^ ^]^ However, this method lacks selectivity as the salt solution spreads extensively throughout the porous brown part due to capillary forces. Ouyang et al. demonstrated the preparation of luminescent glasses with a controlled distribution of dopants such as Eu^3+^, Ce^3+^, Tb^3+^ and Pr^3+^ by using different doped and non‐doped printing resins, which were exchanged during the print using a print‐stop‐print strategy.^[^ ^38^ ^]^ Using this approach, doped silicate glass layers were created with a 100 µm resolution, which show photoinduced features under UV illumination. However, this approach is highly time‐consuming due to the print‐stop‐print process, particularly in fabrication of multilayer structures, as the parts have to be carefully washed after every step. Further, due to vat‐exchange during the process, dopant incorporation is limited to a single dopant per layer. Using DIW, some first gradient refractive index lenses where fabricated by printing a silica ink and a second silica ink containing TiO_2_.^[^ ^39^ ^]^ A major limitation of this approach is its low resolution and rough surface requiring mechanical post‐processing to achieve an optical quality surface. In addition, the method does not allow strict separation between two phases, consequently always resulting in a gradient in composition. Wang et al. presented a method for fabricating alumina‐doped GRIN lenses by selectively depositing an aluminum nitrate solution onto silica powder layers via binder jetting.^[^ ^40^ ^]^ This method is however time‐consuming and leads to migration of the dopant during printing that causes concentration deviations.

While dopant integration was shown, none of the previously presented methods allows for high‐resolution, 3D glass modification within a freely shaped glass body, which is essential for creating precise, integrated optics. In this work, we demonstrate for the first time that the material properties of 3D shaped fused silica glass can be locally modified in 3D with single micron precision in a single doping step. This allows additional functions or anti‐counterfeiting features to be integrated directly within previously shaped glasses. For this purpose, liquid or thermoplastic silica nanocomposites are first shaped by UV‐casting, vat photopolymerization or IM. Consecutively, the organic binder matrix is removed, resulting in porous brown parts. The brown parts are then infused with a photocurable material consisting of a monofunctional methacrylate, a photoinitiator, a solvent and metal oxide precursors. The infused parts are subsequently illuminated using high‐resolution shaping technologies such as lithography, TPL or volumetric printing methods like CAL. The non‐polymerized precursor mixture is removed by developing in a solvent mixture, and the brown part is transformed into a fully dense glass by thermal debinding and subsequent sintering. We demonstrate that using this technology, properties like color, luminescence and refractive index can be changed by locally integrating transition metal ions or rare earth metal ions with micron resolution. In addition to single dopants, this method enables co‐doping of multiple ions at arbitrary positions within the glass. We further demonstrate a scalable process for mass customization through simultaneous doping of 80 parts.

## 2 Results and Discussion

**Figure 1:**
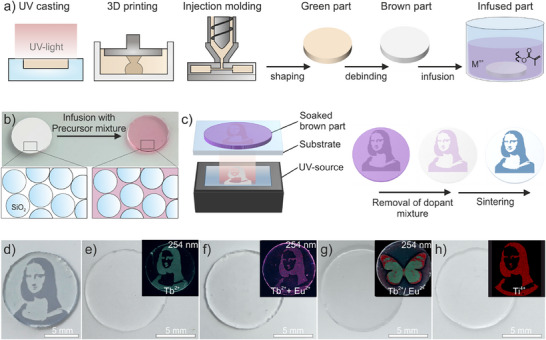
Selective doping of nanocomposite‐derived silicate glasses by light‐induced curing of photocurable precursor mixtures. a) Porous brown parts for subsequent modification can be obtained by shaping nanocomposites using UV‐casting, vat photopolymerization, or IM followed by thermal debinding. An acrylate‐based mixture with metal oxide precursors is used to infuse the brown parts prior to their modification. b) A transparent component is obtained through the infusion of the brown part with a Co^2+^ containing material that matches the refractive index of the silica brown part. c) The precursor mixture within the brown part is then locally cured using UV‐light. The uncured precursor mixture is then removed using a suitable solvent. Afterwards, the parts are debinded and sintered to full density. d) Glass doped with a Co^2+^ mixture showing a blue colored Mona Lisa. e) Image of a glass doped with a Tb^3^⁺, showing blue to green fluorescence under UV‐illumination. f) Image of a glass doped with a mixture of Tb^3+^ and Eu^3+^. Doping with the two salts leads to a purple coloration under UV‐light. g) Image of a glass showing a two‐colored butterfly under UV‐light prepared by co‐doping separate areas with Eu^+3^ and Tb^3+^. h) Image of a glass doped with a Ti^4+^ precursor as well as an X‐ray fluorescence (XRF) image of the doped region.

The general process of glass doping is shown in **Figure** **1**. Silica nanocomposites are shaped using UV‐casting, 3D printing or injection molding followed by thermal debinding to remove the organic binder matrix, resulting in porous brown parts (Figure 1a). Since the nanocomposites used in this work contain 50 vol% organic binder, the resulting brown parts exhibit a porosity of ∼50 vol% after debinding. The porous parts are subsequently infused using a photocurable precursor mixture. Different metal salts were chosen to create different effects in the final glass: To create luminescent sections in the glass, metal salts of Eu^3+^ and Tb^3+^ were used, while Co^2+^ was chosen to create a glass with intense blue coloration. Ti^4+^ doping was used to locally increase the refractive index of the glass. To achieve the integration of the dopants, Eu^3+^, Tb^3+^ and Co^2+^ metal salts were dissolved in an acrylate‐based, photocurable resin, while Ti^4+^ was integrated by the addition of an alkoxide‐based, photocurable precursor.^[^ ^32^ ^]^ The dopant mixtures were adapted to the refractive index of the silica nanoparticles in order to obtain transparent parts when infusing the silica brown part with the dopant mixtures. Table S1 (Supporting Information) shows a comparison of the refractive indices of the dopant mixtures and silica. This allows the light to penetrate the infused parts for curing at arbitrary positions within the bulk (Figure 1b). Selective illumination induces local polymerization, thereby immobilizing the metal oxide precursor in the intended area. For illustration of the process, the dopant mixtures were selectively polymerized using a monochrome LCD display to mask a UV‐light source (Figure 1c). Subsequently, the parts were developed by removing uncured material in a mixture of isopropanol and propionic acid. The addition of propionic acid increases the solubility of the dopants in the developer, enabling significantly faster and more efficient development. Following debinding and sintering, locally doped and transparent glass components were obtained. As shown in Figure 1d, Co^2^⁺ was successfully incorporated into the glass using this method, resulting in a blue coloration of the doped areas and revealing an image of the Mona Lisa. Doping with Tb^3^⁺ yielded a glass that exhibits fluorescence under UV light (Figure 1e). In addition to single dopants, mixtures of dopants can be used, for example, to generate new colors. Figure 1f shows that co‐doping with Tb^3^⁺ and Eu^3^⁺ results in the generation of purple fluorescence. Moreover, by sequentially infusing and exposing the brown parts with different dopant mixtures, glasses with differently doped regions can be fabricated. As shown in Figure 1g, a two‐colored butterfly was created by selectively doping with Tb^3^⁺ and Eu^3^⁺. In addition to the generation of colors or luminescence, the optical properties can also be modified by the addition of Ti^4+^.^[^ ^32^ ^]^ The XRF measurement shown in Figure 1h confirms the incorporation of 3 wt% Ti⁴⁺ to locally increase the refractive index of the glass.^[^ ^32^ ^]^ A measurement of the refractive index n_D_ of 1.4754 ± 0.0006 confirms an increase relative to pure fused silica with a n_D_ of 1.4584.^[^ ^41^ ^]^ To increase the range of usable precursors, an inverse process was also designed. Some highly soluble salts, such as gold chloride could in principle be used in this process, however, they are removed during the development process. To avoid this, brown parts are infused with an acrylate mixture and locally cured as described above. After removing the unreacted acrylate mixture, the brown parts are completely immersed into a dopant solution. The cured part of the sample now prevents the dopant from reaching this specific area in the brown part. After thermal removal of the organic binder, the part is sintered to full density. Besides a Co^2^⁺‐doped glass, an Au‐nanoparticle doped glass, so far not achievable by direct selective doping, was fabricated via the inverse approach (Figure S1, Supporting Information).

Especially for optical applications, maintaining high transmittance is crucial. UV‐Vis spectroscopy confirmed the high transmission of the doped glasses for the dopant concentrations used in this study (Figure S2, Supporting Information). Furthermore, accurate calculation of shrinkage is essential for determining the exact final dimensions of the doped regions and glasses, as doped areas contain a slightly higher solid loading. Figure S3a (Supporting Information) demonstrates that the shrinkage of a Co^2+^ doped glass sample is isotropic and can therefore be calculated as a function of the solid loading of the brown parts.^[^ ^17^, ^32^ ^]^ Besides doping with small quantities of Co^2+^, the incorporation of 3 wt% Ti⁴⁺ (Table S2, Supporting Information), which significantly increases the solid loading of the brown part, was analyzed. Figure S3b (Supporting Information) shows, that doping with 3 wt% Ti^4+^ did not result in a significant variation in the shrinkage calculated from the solid loading of the brown parts.

**Figure 2:**
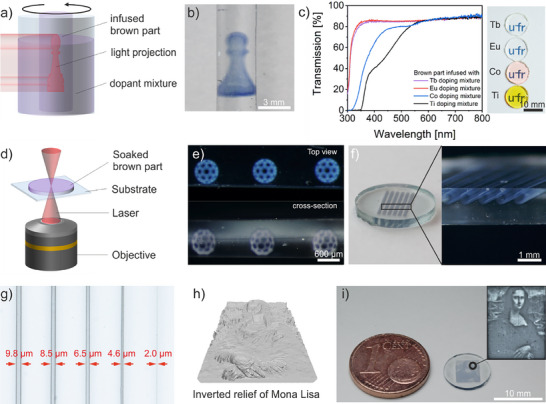
3D selective doping within silicate glasses. a) Conceptual illustration of 3D doping within a porous glass rod using CAL. The infused brown part is placed within a vial containing the precursor mixture for selective illumination. b) Co^2+^ doped pawn printed inside a glass rod using CAL. c) Transmission spectrum of brown parts with a thickness of 1 mm infused with precursors of Tb^3+^, Eu^3+^, Co^3+^ and Ti^4+^ next to images of the analyzed brown parts. d) Schematic illustration of selective doping using TPL. e) 3D bucky balls created by doping with Co^2+^. f) Co^2+^ doped hollow microtubes in glass. g) Resolution of Co^2+^ doping in the single micron range. Structures down to 2.0 µm were achieved. h) Visualization of an inverse relief used to print the Mona Lisa in glass. By varying the thickness, areas of different shades are created. i) Portrait of Mona Lisa in glass generated by printing the relief in h).

Compared to 2.5‐dimensional lithographic doping as shown in Figure 1, methods such as CAL and TPL enable 3D polymerization of the precursor mixtures at arbitrary positions within the brown part resulting in a 3D doping of the glass. CAL is a volumetric 3D printing technique in which a photosensitive resin is cured inside a rotating vial by a series of 2D light projections.^[^ ^22^, ^42^, ^43^ ^]^ Due to the superposition of the projections, the exposure dose exceeds the polymerization threshold only in desired regions, enabling selective curing of the resin. So far the technology has mainly been used to shape polymers as well as materials, which can be subsequently converted glass or ceramics.^[^ ^22^, ^44^, ^45^ ^]^ In this work we demonstrate that this technology can also be used to integrate the dopant locally in 3D. For doping within the glass using CAL, the brown part is initially infused with the precursor mixture and subsequently placed in a vial containing the same precursor mixture (**Figure** **2**a). A 3D structure is then printed into the brown part using a LCD‐based CAL system.^[^ ^46^ ^]^ After subsequent development and thermal treatment, a structure completely embedded in fused silica glass is obtained (Figure 2b). Exposure and polymerization through several millimeters in thickness is facilitated by the high transmission of the infused brown parts, as shown in Figure 2c.

In particular, for the fabrication of integrated optics, it is crucial to achieve high‐resolution doping. TPL offers significantly higher resolution for the creation of 3D structures within glass than CAL. TPL is based on the simultaneous absorption of multiple photons in a tightly focused laser spot, which initiates polymerization within the focal volume only.^[^ ^47^ ^]^ For doping via TPL, the laser of a commercial printer is focused within the transparent, infused brown part, as can be seen in Figure 2d. Figure 2e,f shows bucky balls and microtubes with several millimeters in length demonstrating the capability to create complex 3D microstructures inside the glass. A particular strength of TPL is its exceptionally high printing resolution. As can be seen in Figure 2g, TPL enables local doping with single‐digit micrometer precision, allowing the creation of highly detailed doped regions. The high resolution of TPL in combination with a 3D design enables the generation of highly complex images, which can be useful, for example, for the permanent 3D storage of information in glass.^[^ ^48^ ^]^ Figure 2h,i showcases this through the fabrication of a 3D portrait of the Mona Lisa. By controlling the doping thickness within the glass, regions with different intensities of coloration can be produced, enabling the creation of highly detailed images.

**Figure 3:**
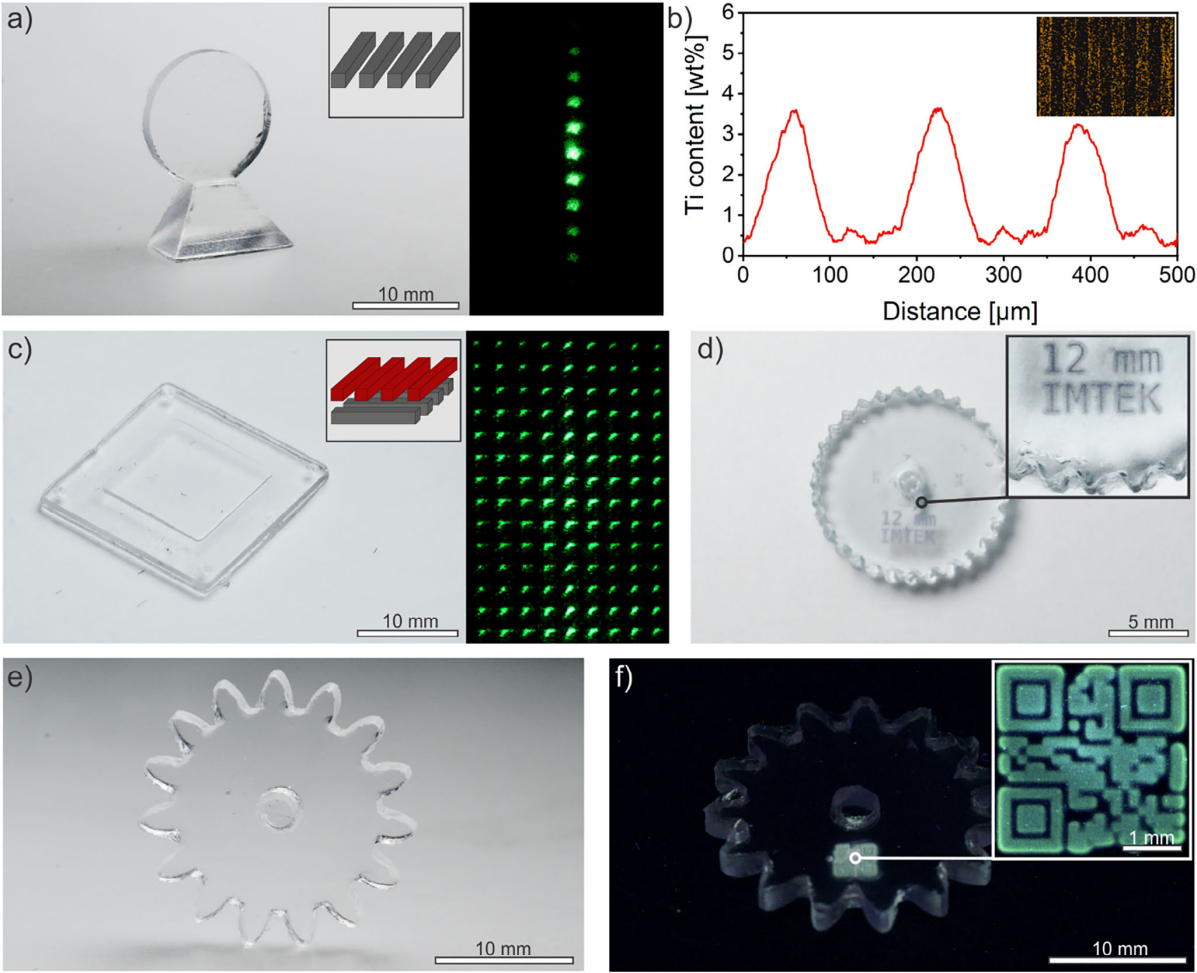
Modification of UV‐casted and 3D‐printed fused silica components using TPL. a) 3D‐printed glass component into which a DOE consisting of Ti^4+^ doped lines with a diameter of 100 µm was incorporated as well as the corresponding projection pattern. b) EDX line measurement of the Ti^4+^ content in the DOE in a). An EDX mapping can be seen in the inset. c) Two stacked Ti^4+^ doped line DOEs with a thickness of 100 µm which are perpendicular to each other with a 200 µm spacing as well as the projection pattern. Doping was carried out within a component featuring an alignment frame for optical setups. d) 3D‐printed gear showing a permanent labeling through Co^2+^ doping. e) UV‐casted gear that has been made traceable and counterfeit‐proof with a fluorescent Tb^3+^ doped QR code. No doping is visible in natural light. f) Gear wheel from e) under UV‐light of 254 nm. The inset shows a magnified image of the QR code visible under UV‐light.

Using the selective doping method presented here, additional functions can be added to the final glasses after shaping of the nanocomposites. For instance, the refractive index of silicate glasses can be increased by incorporating metal oxides such as TiO_2_.^[^ ^49^, ^50^, ^51^ ^]^ The ability to locally increase the refractive index allows optical components such as a diffractive optical element (DOE) to be integrated directly into the glass components. Vat photopolymerization allowed to shape the geometry to directly integrate holding elements (**Figure** **3**a) or distance spacers onto the optical component significantly simplifying optical setups. The difference in the Ti^4+^ concentration in the DOE is confirmed by EDX line measurement as can be seen in Figure 3b.

To improve diffraction efficiency, stacking multiple DOEs can be beneficial.^[^ ^52^, ^53^ ^]^ With the method presented, creation of stacked, precisely pre‐aligned DOEs within one part is possible due to freeform design. Figure 3c shows exemplary two linear DOEs printed perpendicular to each other with a distance of 200 µm. The first layer diffracts light along one axis, while the second layer causes orthogonal diffraction, resulting in a 2D diffraction pattern.

With the increasing prevalence of counterfeiting worldwide, the integration of watermarks or security elements offers an important form of product protection.^[^ ^54^, ^55^, ^56^ ^]^ As illustrated in Figure 3d, TPL can also be used for permanent product labeling that persist even under harsh conditions such as heat or in contact with chemicals that do not attack the surrounding glass. Furthermore, glass components can be marked with a fluorescent QR code to prevent counterfeiting or to enable traceability (Figure 3e,f).

**Figure 4:**
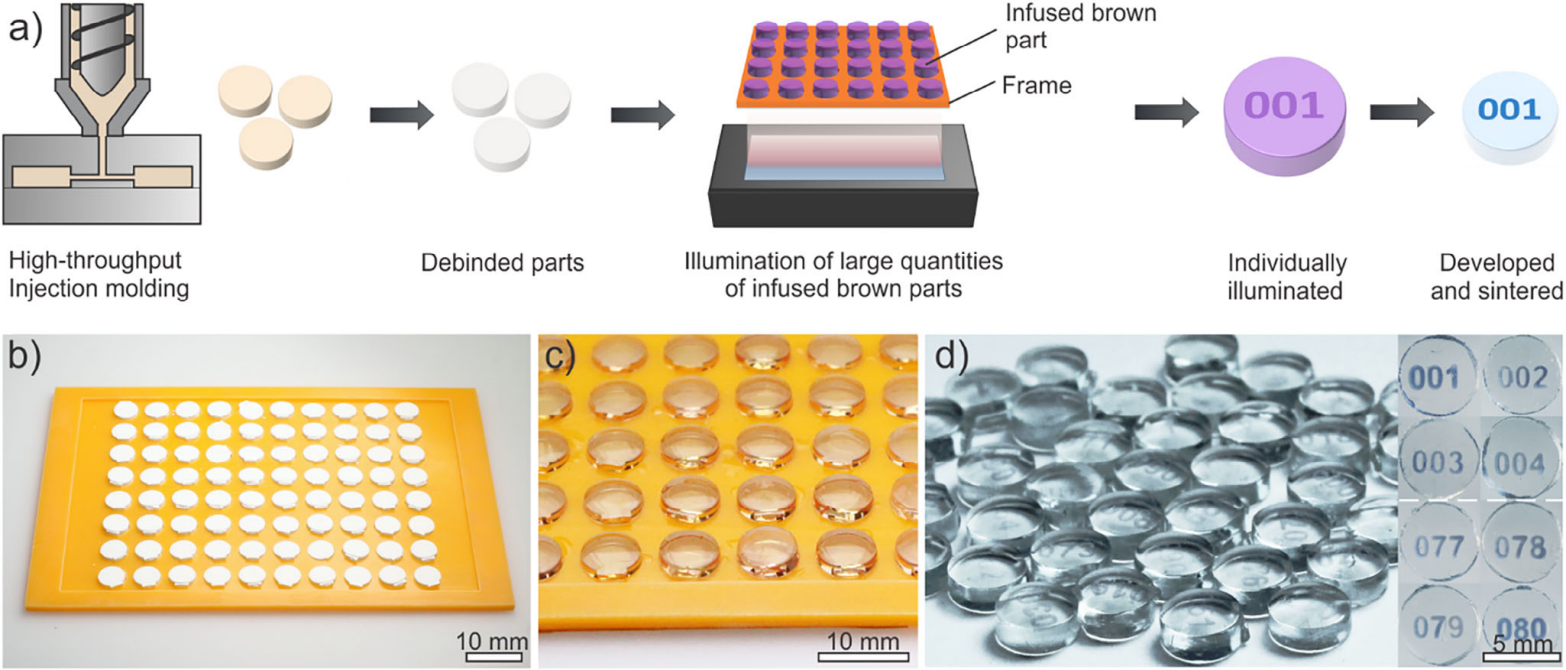
Mass customization of injection‐molded brown parts and their conversion into individually doped glass parts. a) Principle of mass customization of injection‐molded parts. After thermal conversion to porous brown parts, the injection‐molded parts are placed in a rack with defined spacing and infused with a precursor mixture. All parts are individually illuminated at the same time. Subsequently, the parts are converted into dense doped glasses. b) 80 porous brown parts and c) infused brown parts in a 3D‐printed rack which defines the exact position. d) Individually doped glass after sintering showing unique labels. The first four and last four numbers of the series are highlighted on the right side.

Of particular interest is the combination of methods that offer both high scalability and a high degree of customization. Mass production processes such as injection molding of silica nanocomposites enable the production of glass components in large quantities with short cycle times.^[^ ^23^ ^]^ However, it is not suitable to customize parts, as each design requires an individual tool. While injection molding alone is not suitable for individualized glass production, its combination with localized, lithography‐based doping enables the efficient customization of high volumes of glass components. This enables, for example, unique serial numbers or security features to be integrated with high efficiency in a standardized glass shape. **Figure** **4**a shows the principle of mass customization of doped glasses using injection‐molded parts.

First, injection‐molded parts were fabricated and debinded, as previously described.^[^ ^23^ ^]^ The parts were subsequently arranged in a custom‐designed rack to ensure the correct position of the parts during the illumination process (Figure 4b). Following infusion with a Co^2^⁺‐containing dopant precursor (Figure 4c), all parts were illuminated at the same time using a monochrome LCD display. Since the exact position of the parts were predetermined, individual exposure of each part was possible, allowing each component to receive a unique number combination. After subsequent development, debinding and sintering of the parts, individually doped glass components were obtained (Figure 4d). Since components are exposed simultaneously, the processing time is unaffected by batch size, offering potential for high‐throughput industrial use.

## 3 Conclusion

We have developed an innovative technique, demonstrating that by infusing brown parts with a photoactive precursor followed by precise local curing, glass components with local composition modifications can be produced. This allows properties such as color, luminescence or refractive index to be modified locally using the dopants Co^2+^, Tb^3+^, Eu^3+^ and Ti^4+^. Using high‐resolution 2.5 or 3D lithography processes, this approach enables a number of novel applications from integrated optics and photonics to anti‐counterfeiting features and data storage from single 3D printed prototypes to mass customized glass shapes derived from silica injection molding. The possibility of combining different structuring methods with the subsequent modification of properties allows the advantages of high‐throughput methods, such as IM, to be combined with the high precision or customizability of lithography processes.

## 4 Experimental Section

### Materials

All materials were used as supplied. Glassomer V2 glass printing resin, Glassomer developer and Glassomer UV‐casting resin were kindly provided by Glassomer GmbH, Germany. Ethylene glycol, mono‐2‐(methacryloyloxy)ethyl succinate (MAOES), propionic acid, phenylbis(2,4,6‐trimethylbenzoyl)phosphine oxide (BAPO), titanium tetraisopropoxide, isopropanol, cobalt(II) acetylacetonate, europium(III) acetylacetonate hydrate, terbium(III) acetylacetonate hydrate and gold(III) chloride were purchased from Sigma Aldrich, Germany. 2‐((Benzoyloxy)imino)‐1‐(4‐(phenylthio)phenyl)octan‐1‐one (OXE 01) was purchased from abcr, Germany.

### Preparation of the Brown Parts

The Glassomer UV‐casting resin was cured for 1 min on each side using a Superlite 400 (Lumatec, Germany) UV‐light source operating at a wavelength of 300–400 nm. The nanocomposite Glassomer V2 was 3D‐printed using a vat photopolymerization printer type SL1S SPEED (Prusa Research a.s., Czech Republic) according to manufacturer's instructions. The printed parts were then developed for 10 min at room temperature using the Glassomer developer. The injection‐molded brown parts were provided by Glassomer GmbH. The casted and printed parts were then thermally debinded according to the manufacturer's instructions.

### Preparation of the Precursor Mixtures

For the precursor mixture, MAOES and ethylene glycol were mixed in a 4:1 ratio (wt/wt). Salts of Co^2+^ (40 mM), Eu^3+^ (5 mM) or Tb^3+^ (5 mM) were added to the mixture. For Ti^4+^ doped glasses, the precursor described in Prediger et al.^[^ ^32^ ^]^ was prepared and added to the mixture up to a 1:1 (wt/wt) ratio. For the selective curing of the precursor mixtures using the monochrome display of a Prusa SL1S printer, 0.2 wt% BAPO were added while 3 wt% OXE 01 was added for polymerization using TPL. The precursor mixtures were stirred for 1 h at room temperature before usage.

### Infusion of Brown Parts

The brown parts were placed in a reservoir containing the precursor mixture until they were completely soaked. The infusion time ranged from 10 min for samples with a thickness of 1 mm to 30 min for samples with a thickness of 4 mm. Complete infusion was confirmed by the transparency of the parts after absorbing the index‐matched dopant mixture.

### Curing of the Precursor Mixture

Illumination of the brown parts was performed using a monochrome LCD display with 2560 × 1620 pixel of a commercial 3D printer type SL1S SPEED (Prusa Research a.s., Czech Republic). TPL was performed with a 4x or 10x objective on a NanoOne printer (UpNano GmbH, Austria) in top down mode with a laser power of 150–200 mW, a layer thickness of 3 µm and a scanning speed of 600 mm s^−1^ using the 10x objective. A laser power of 200–250 mW, a layer thickness of 5 µm and a scanning speed of 750 mm s^−1^ were selected for the 4x objective. CAL was performed on a LCD‐based printer with a wavelength of 450 nm, an intensity of 20 mW cm^−2^ and a LCD pixel size of 50 µm described by Tisato et al.^[^ ^46^ ^]^ Printing was carried out with a rotational speed of 20°/s and a framerate of 20 fps. Initially, the brown parts were infused with the dopant mixture, placed in a vial containing the same mixture, and then exposed to projection after rotation of the vial was started. After 240 s, projection and rotation were stopped and the parts were gently removed from the vial. Afterwards, uncured resin was removed in an isopropanol/ propionic acid (10 : 1 vol/vol) bath under stirring. Over a period of 12 h, the developer solution was replaced five times to ensure effective removal of the resin.

### Heat Treatment

The decomposition of the cured dopant mixture during thermal treatment was investigated using a TGA type STAA449F5 (Netzsch, Germany). The doped brown parts were thermally debinded at 600 °C using an ashing furnace of type AAF (Carbolite Gero, Germany) to remove the organics and decompose the precursor as shown in Figure S4 (Supporting Information). For subsequent densification, the Co^2+^, Tb^3+^ and Eu^3+^ doped parts were sintered using a furnace type BLF 18/3 (Carbolite Gero, Germany) with a heating rate of 5 °C min^−1^ at a temperature of 1300 °C for 2 h. According to our previous work, the Ti⁴⁺ doped glasses were heated to temperatures up to 1500 °C to obtain an amorphous glass free of the anatase phase.^[^ ^32^ ^]^ The Ti⁴⁺ doped samples were therefore presintered at 1250 °C for 1 h at a pressure of 1 × 10^−3^ mbar with a heating and cooling rate of 3 °C min^−1^ using a vacuum furnace type STF16/450 (Carbolite Gero, Germany). Subsequently, the parts were heated to 1500 °C for 5 min under atmospheric conditions in the BLF 18/3 with heating and cooling rates of 5 °C min^−1^.

### Characterization

The transmission was measured using a UV–vis spectrometer type Evolution 201 (Thermo Scientific, Germany). EDX measurements were carried out using an Octane Elite EDS spectrometer (EDAX, Germany) with a 20 kV acceleration speed.

A M4 TORNADO (Bruker, USA) with a source current of 200 µA, a spot size of 20 µm and a scan rate of 30 ms per point was used for XRF measurements under vacuum to detect the dopants in the glasses. The refractive index n_D_ of the dopant mixtures and the glass were measured using a digital refractometer type AR200 (Reichert, USA) at wavelength of 589.3 nm. The measurements were carried out in an air‐conditioned laboratory with a constant temperature of 20 °C. Prior to the measurement, the refractometer and samples were stored in the temperature‐controlled room for 24 h. For each sample, five measurements were carried out and the mean value was calculated.

## Conflict of Interest

Glassomer GmbH has patented the technology described within this paper (application no. PCT/EP2022/058116) and is in the process of commercializing it. The authors declare no other conflict of interest.

## Author Contributions

F.K‐H. conceived the idea. R.P. designed and conducted the experiments. R.P., J.N.H., and L.H. analyzed the materials. S.K. supported in the preparation of brown parts and sintering. S.T. performed CAL experiments. All authors contributed to writing the manuscript.

## Supporting information



Supporting Information

## Data Availability

The data that support the findings of this study are available in the supplementary material of this article.

## References

[1] H. G. Pfaender , Schott Guide to Glass, Springer, Netherlands, Dordrecht, 1996.

[2] D. L. Morse , J. W. Evenson , International Journal of Applied Glass Science 2016, 7, 409.

[3] B. E. Rapp , F. Kotz‐Helmer , Additive Manufacturing of Glass: From Science to Applications, Elsevier, Amsterdam 2024.

[4] J. Zhang , M. Gecevičius , M. Beresna , P. G. Kazansky , Phys. Rev. Lett. 2014, 112, 033901. 24484138 10.1103/PhysRevLett.112.033901

[5] M. Djamal , L. Yuliantini , R. Hidayat , K. Boonin , P. Yasaka , J. Kaewkhao , Mater. Today: Proc. 2018, 5, 15126.

[6] R. Kingslake , R. B. Johnson , Lens Design Fundamentals, Academic Press, San Diego, CA 2009.

[7] P. Hartmann , R. Jedamzik , S. Reichel , B. Schreder , Appl. Opt. 2010, 49, D157.

[8] Z. Zhang , J. Yan , T. Kuriyagawa , Int. J. Extrem. Manuf. 2019, 1, 022001.

[9] X. Luo , L. Lu , M. Yin , X. Fang , X. Chen , D. Li , L. Yang , G. Li , J. Ma , Mater. Res. Bull. 2019, 109, 183.

[10] H. Park , S. M. Iftiquar , M. Shin , H. Kim , J. Jung , S. Kim , A. H. T. Le , Y. Kim , D. P. Pham , J.‐S. Jeong , J. Yi , JPE 2017, 7, 025502.

[11] A. Waldbaur , H. Rapp , K. Länge , B. E. Rapp , Anal. Methods 2011, 3, 2681.

[12] C. W. Ponader , J. F. Schroeder , A. M. Streltsov , J. Appl. Phys. 2008, 103, 063516.

[13] V. R. Bhardwaj , E. Simova , P. B. Corkum , D. M. Rayner , C. Hnatovsky , R. S. Taylor , B. Schreder , M. Kluge , J. Zimmer , J. Appl. Phys. 2005, 97, 083102.

[14] D. Hülsenberg , A. Harnisch , A. Bismarck , Microstructuring of Glasses, Springer, Berlin New York 2008.

[15] F. Kotz , K. Plewa , W. Bauer , N. Schneider , N. Keller , T. Nargang , D. Helmer , K. Sachsenheimer , M. Schäfer , M. Worgull , C. Greiner , C. Richter , B. E. Rapp , Adv. Mater. 2016, 28, 4646. 27060964 10.1002/adma.201506089

[16] F. Kotz , P. Risch , K. Arnold , S. Sevim , J. Puigmartí‐Luis , A. Quick , M. Thiel , A. Hrynevich , P. D. Dalton , D. Helmer , B. E. Rapp , Nat. Commun. 2019, 10, 1439. 30926801 10.1038/s41467-019-09497-z PMC6441035

[17] F. Kotz , K. Arnold , W. Bauer , D. Schild , N. Keller , K. Sachsenheimer , T. M. Nargang , C. Richter , D. Helmer , B. E. Rapp , Nature 2017, 544, 337. 28425999 10.1038/nature22061

[18] Z. Li , Y. Jia , K. Duan , R. Xiao , J. Qiao , S. Liang , S. Wang , J. Chen , H. Wu , Y. Lu , X. Wen , Nat. Commun. 2024, 15, 2689. 38538612 10.1038/s41467-024-46929-x PMC10973333

[19] F. Kotz , A. S. Quick , P. Risch , T. Martin , T. Hoose , M. Thiel , D. Helmer , B. E. Rapp , Adv. Mater. 2021, 33, 2006341. 33448090 10.1002/adma.202006341 PMC11469267

[20] X. Wen , B. Zhang , W. Wang , F. Ye , S. Yue , H. Guo , G. Gao , Y. Zhao , Q. Fang , C. Nguyen , X. Zhang , J. Bao , J. T. Robinson , P. M. Ajayan , J. Lou , Nat. Mater. 2021, 20, 1506. 34650230 10.1038/s41563-021-01111-2

[21] M. Mader , L. Hambitzer , P. Schlautmann , S. Jenne , C. Greiner , F. Hirth , D. Helmer , F. Kotz‐Helmer , B. E. Rapp , Adv. Sci. 2021, 8, 2103180. 10.1002/advs.202103180 PMC8655167 34668342

[22] J. T. Toombs , M. Luitz , C. C. Cook , S. Jenne , C. C. Li , B. E. Rapp , F. Kotz‐Helmer , H. K. Taylor , Science 2022, 376, 308. 35420940 10.1126/science.abm6459

[23] M. Mader , O. Schlatter , B. Heck , A. Warmbold , A. Dorn , H. Zappe , P. Risch , D. Helmer , F. Kotz , B. E. Rapp , Science 2021, 372, 182. 33833122 10.1126/science.abf1537

[24] S. Guo , M. A. Ali , M. A. Mohamed , X. Han , X. Liu , J. Qiu , Mater. Chem. Front. 2024, 8, 1400.

[25] I. Cooperstein , E. Shukrun , O. Press , A. Kamyshny , S. Magdassi , ACS Appl. Mater. Interfaces 2018, 10, 18879. 29741081 10.1021/acsami.8b03766

[26] D. T. Nguyen , C. Meyers , T. D. Yee , N. A. Dudukovic , J. F. Destino , C. Zhu , E. B. Duoss , T. F. Baumann , T. Suratwala , J. E. Smay , R. Dylla‐Spears , Adv. Mater. 2017, 29, 1701181. 10.1002/adma.201701181 28452163

[27] A. De Marzi , G. Giometti , J. Erler , P. Colombo , G. Franchin , Addit. Manuf. 2022, 54, 102727.

[28] Z. Hong , P. Ye , D. A. Loy , D. A. Loy , D. A. Loy , R. Liang , Optica 2021, 8, 904.

[29] J. Bauer , C. Crook , T. Baldacchini , Science 2023, 380, 960. 37262172 10.1126/science.abq3037

[30] P.‐H. Huang , M. Laakso , P. Edinger , O. Hartwig , G. S. Duesberg , L.‐L. Lai , J. Mayer , J. Nyman , C. Errando‐Herranz , G. Stemme , K. B. Gylfason , F. Niklaus , Nat. Commun. 2023, 14, 3305. 37280208 10.1038/s41467-023-38996-3 PMC10244462

[31] M. Li , L. Yue , A. C. Rajan , L. Yu , H. Sahu , S. M. Montgomery , R. Ramprasad , H. J. Qi , Sci. Adv. 2023, 9, adi2958. 10.1126/sciadv.adi2958 PMC10550221 37792949

[32] R. Prediger , S. Kluck , L. Hambitzer , D. Sauter , F. Kotz‐Helmer , Adv. Mater. 2024, 36, 2407630. 10.1002/adma.202407630 39219207

[33] D. G. Moore , L. Barbera , K. Masania , A. R. Studart , Nat. Mater. 2020, 19, 212. 31712744 10.1038/s41563-019-0525-y

[34] B. Li , Z. Li , I. Cooperstein , W. Shan , S. Wang , B. Jiang , L. Zhang , S. Magdassi , J. He , Adv. Sci. 2023, 10, 2305775. 10.1002/advs.202305775 PMC10724418 37870213

[35] K. Sasan , A. Lange , T. D. Yee , N. Dudukovic , D. T. Nguyen , M. A. Johnson , O. D. Herrera , J. H. Yoo , A. M. Sawvel , M. E. Ellis , C. M. Mah , R. Ryerson , L. L. Wong , T. Suratwala , J. F. Destino , R. Dylla‐Spears , ACS Appl. Mater. Interfaces 2020, 12, 6736. 31934741 10.1021/acsami.9b21136

[36] A. C. Chinn , E. L. Marsh , T. Nguyen , Z. B. Alhejaj , M. J. Butler , B. T. Nguyen , K. Sasan , R. J. Dylla‐Spears , J. F. Destino , ACS omega 2022, 7, 17492. 35647440 10.1021/acsomega.2c02292 PMC9134392

[37] C. Liu , B. Qian , R. Ni , X. Liu , J. Qiu , RSC Adv. 2018, 8, 31564. 35548226 10.1039/c8ra06706f PMC9085626

[38] M. Ouyang , H. Zhang , M. Li , J. Zhang , Y. Gong , X. Huang , X. Liu , J. Qiu , Z. Yang , G. Dong , Laser Photonics Rev. 2023, 17, 2300068.

[39] R. Dylla‐Spears , T. D. Yee , K. Sasan , D. T. Nguyen , N. A. Dudukovic , J. M. Ortega , M. A. Johnson , O. D. Herrera , F. J. Ryerson , L. L. Wong , Sci. Adv. 2020, 6, abc7429. 10.1126/sciadv.abc7429 PMC7673801 33208366

[40] H.‐R. Wang , M. J. Cima , B. D. Kernan , E. M. Sachs , J. Non‐Cryst. Solids 2004, 349, 360.

[41] I. H. Malitson , J. Opt. Soc. Am. 1965, 55, 1205.

[42] B. E. Kelly , I. Bhattacharya , H. Heidari , M. Shusteff , C. M. Spadaccini , H. K. Taylor , Science 2019, 363, 1075. 30705152 10.1126/science.aau7114

[43] P. N. Bernal , P. Delrot , D. Loterie , Y. Li , J. Malda , C. Moser , R. Levato , Adv. Mater. 2019, 31, 1904209. 10.1002/adma.201904209 31423698

[44] M. Kollep , G. Konstantinou , J. Madrid‐Wolff , A. Boniface , L. Hagelüken , P. V. W. Sasikumar , G. Blugan , P. Delrot , D. Loterie , J. Brugger , C. Moser , Adv. Eng. Mater. 2022, 24, 2101345.

[45] J. Madrid‐Wolff , J. Toombs , R. Rizzo , P. N. Bernal , D. Porcincula , R. Walton , B. Wang , F. Kotz‐Helmer , Y. Yang , D. Kaplan , Y. S. Zhang , M. Zenobi‐Wong , R. R. McLeod , B. Rapp , J. Schwartz , M. Shusteff , H. Talyor , R. Levato , C. Moser , MRS Commun. 2023, 13, 764. 37901477 10.1557/s43579-023-00447-x PMC10600040

[46] S. Tisato , G. Vera , A. Mani , T. Chase , D. Helmer , Addit. Manuf. 2024, 87, 104232.

[47] X. Zhou , Y. Hou , J. Lin , AIP Adv. 2015, 5, 030701.

[48] Z. Hu , X. Huang , Z. Yang , J. Qiu , Z. Song , J. Zhang , G. Dong , Light Sci. Appl. 2021, 10, 140. 34234097 10.1038/s41377-021-00581-y PMC8263721

[49] G. Scannell , S. Barra , L. Huang , J. Non‐Cryst. Solids 2016, 448, 52.

[50] P. C. Schultz , J. Am. Ceram. Soc. 1976, 59, 214.

[51] S. Satoh , K. Susa , I. Matsuyama , J. Non‐Cryst. Solids 1992, 146, 121.

[52] K. Spariosu , I. Tengara , T. P. Jannson , SPIE 1997, 3133, 101.

[53] M. L. Ng , D. Chanda , P. R. Herman , Opt. Express 2012, 20, 23960. 23188362 10.1364/OE.20.023960

[54] X. Yu , H. Zhang , J. Yu , Aggregate 2021, 2, 20.

[55] W. Hong , Z. Yuan , X. Chen , Small 2020, 16, 1907626. 10.1002/smll.201907626 32187853

[56] Y. Huo , Z. Yang , T. Wilson , C. Jiang , Adv. Mater. Interfaces 2022, 9, 2200201.

