# Supplementary material for: High‐Precision 3D Doping of Fused Silica Glass Derived from Nanocomposites
Source: Adv Mater. 2025 Aug 3;37(45):e11245. doi: 10.1002/adma.202511245 (PMC12617063; doi:10.1002/adma.202511245)
Supplement: Supplementary file 1 — Supporting Information [file ADMA-37-e11245-s001.docx]

Supplementary information

**High-Precision Three-Dimensional Doping of Fused Silica Glass Derived from Nanocomposites**

*Richard Prediger, Sebastian Kluck, Leonhard Hambitzer, Bastian E. Rapp, Silvio Tisato, Josephine N. Häberlein, Dorothea Helmer, Frederik Kotz-Helmer^*^*


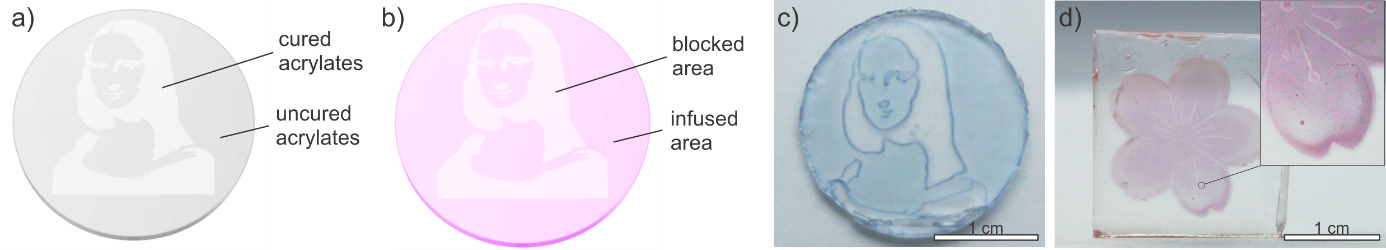


**Figure S1.** Inverse process for doping with highly soluble dopants. a) Selective curing of the acrylate mixture without dopants results in certain areas being covered by polymerized material. The uncured acrylate mixture is then removed by developing the part. b) During the subsequent soaking with a dopant mixture, the covered areas are not infused and therefore doped. c) Co^2+^-doped glass showing a Mona Lisa prepared by the method described. d) Au-doped glass showing pink cherry blossom.


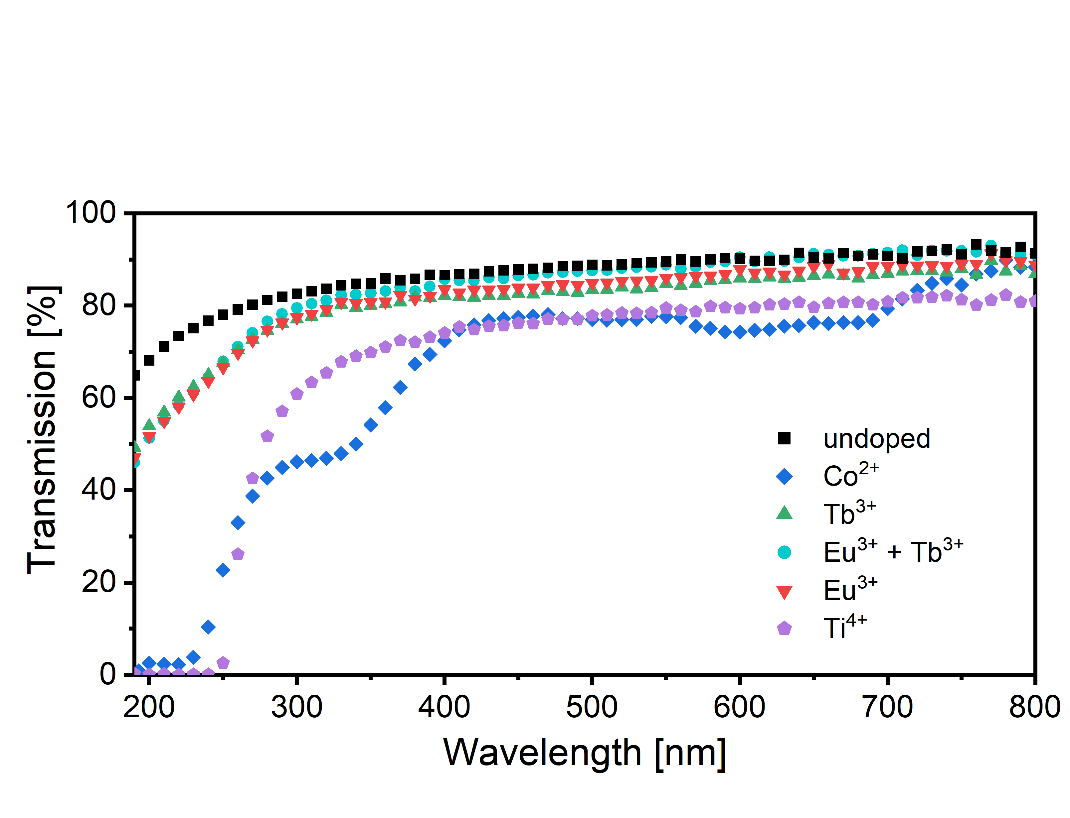


**Figure S2.** UV-Vis transmission measurements of doped glasses with a thickness of 1 mm. A fused silica glass sample without doping served as a reference. The rare earth doped glasses show a comparably high transmission at wavelengths higher 300 nm as the undoped fused silica glass.

**
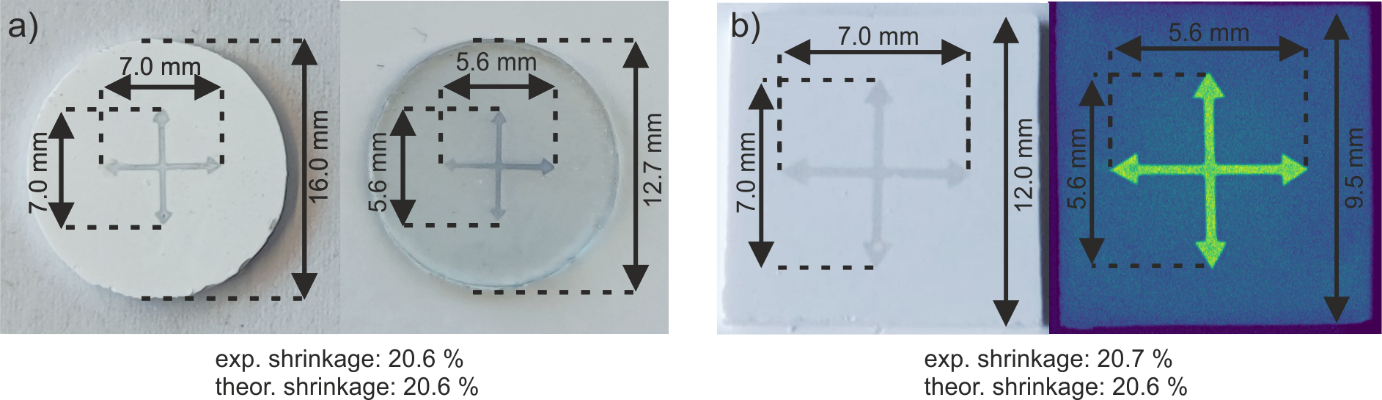
**

**Figure S3.** Determination of linear shrinkage during sintering. a) Brown part (left) and doped glass (right) after selective doping with the Co^2+^ precursor. The experimental shrinkage of 20.6 % matches the theoretical shrinkage. b) Brown part (left) and XRF image of doped glass (right) after selective doping with the Ti^4+^ precursor. The concentration of Ti^4+^ is 3 wt%. Despite the high Ti^4+^ content, the experimental shrinkage of 20.7 % is in good agreement with the theoretical shrinkage of 20.6 %.

**
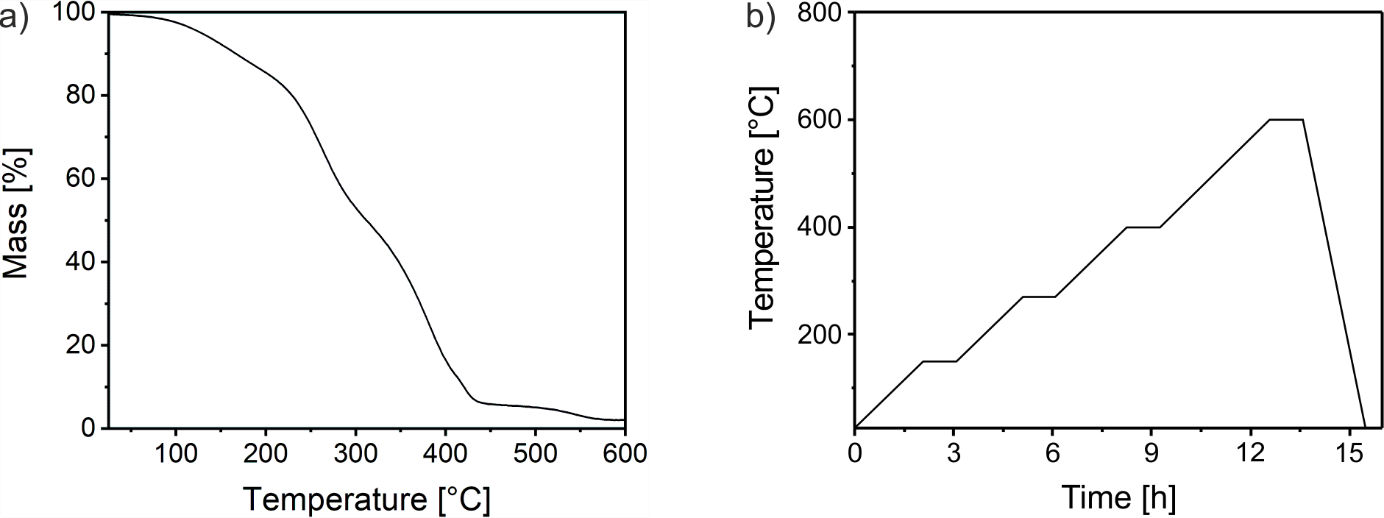
**

**Figure S4**. Optimization of the debinding process for doped brown parts. a) TGA of the pure cured precursor mixture containing 2 wt% europium acetylacetonate. b) Debinding protocol based on the TGA measurement in a).

**Table S1.** Refractive index $n_{D}$ measurement of dopant mixtures for incorporation of Co^2+^, Eu^3+^, Tb^3+^ or Ti^4+^. The mixtures contained salts for Co^2+^ (40 mM), Eu^3+^ (5 mM) or Tb^3+^ (5 mM) doping. The mixture for Ti^4+^ integration consisted of a 1:1 (wt/wt) ratio of the acrylate mixture and the precursor. The refractive index of SiO_2_ serves as a reference.

| **No.** | **SiO_2_**^[41]^ | **Co-doped** | **Tb-doped** | **Eu-doped** | **Ti-doped** |
| --- | --- | --- | --- | --- | --- |
| 1 | 1.4584 | 1.4598 | 1.4597 | 1.4596 | 1.4568 |
| 2 | 1.4584 | 1.4599 | 1.4597 | 1.4597 | 1.4568 |
| 3 | 1.4584 | 1.4599 | 1.4598 | 1.4597 | 1.4568 |
| 4 | 1.4584 | 1.4600 | 1.4597 | 1.4596 | 1.4567 |
| 5 | 1.4584 | 1.4600 | 1.4598 | 1.4596 | 1.4568 |
| **Mean:** | 1.4584 | 1.4599 ± 0.0001 | 1.4597 ± 0.0001 | 1.4596 ± 0.0001 | 1.4568 ± 0.0001 |

**Table S2.** EDX measurement to determine the Ti^4+^ concentration in the measured pattern in Fig. S3. The measurement confirms the calculated concentration of Ti^4+^ of 3 wt%.

| **Element** | **Weight %** | **Atomic %** | **Error %** |
| --- | --- | --- | --- |
| O | 47.8 | 62.2 | 10.2 |
| Si | 49.3 | 36.5 | 4.6 |
| Ti | 3.0 | 1.3 | 5.5 |
